# The Dual Antimelanogenic and Antioxidant Activities of the Essential Oil Extracted from the Leaves of *Acorus macrospadiceus* (Yamamoto) F. N. Wei et Y. K. Li

**DOI:** 10.1155/2012/781280

**Published:** 2012-10-31

**Authors:** Huey-Chun Huang, Hsiao-Fen Wang, Kuang-Hway Yih, Long-Zen Chang, Tsong-Min Chang

**Affiliations:** ^1^Department of Medical Laboratory Science and Biotechnology, China Medical University, No. 91 Hsueh-Shih Road, Taichung 40402, Taiwan; ^2^Department of Hair Styling and Design, Hung Kuang University, No. 34, Chung-Chie Rd., Shalu, Taichung City 43302, Taiwan; ^3^Department of Applied Cosmetology & Master Program of Cosmetic Science, Hung Kuang University, No. 34, Chung-Chie Rd., Shalu, Taichung City 43302, Taiwan; ^4^Crop production Section, General of Agriculture Bureau of Taichung City, Taichung, No. 89, Sec 2, Taichung Port Road, Xitun District, Taichung City 40701, Taiwan

## Abstract

The antimelanogenic and antioxidant activities of the essential oil extracted from the leaves of *Acorus macrospadiceus* (Yamamoto) F. N. Wei et Y. K. Li have never been explored. The essential oil effectively inhibited mushroom tyrosinase activity (EC_50_ = 1.57 mg/mL) and B16F10 tyrosinase activity (IC_50_ = 1.01 mg/mL), decreased the melanin content (EC_50_ = 1.04 mg/mL), and depleted the cellular level of the reactive oxygen species (ROS) (EC_50_ = 1.87 mg/mL). The essential oil effectively scavenged 2,2-diphenyl-1-picryl-hydrazyl (DPPH) (EC_50_ = 0.121 mg/mL) and 2,2′-azino-bis (3-ethylbenzthiazoline-6-sulphonic acid) ABTS^+^ radicals (EC_50_ = 0.122 mg/mL). It also exhibited an apparent reducing power (EC_50_ = 0.021 mg/mL) and metal-ion chelating activity (EC_50_ = 0.029 mg/mL). The chemical constituents of the essential oil are ethers (55.73%), ketones (19.57%), monoterpenes (7.82%), alcohols (3.85%), esters (3.77%), sesquiterpenes (3.72%), and aromatic compounds (2.85%). The results confirm that *A. macrospadiceus* essential oil is a natural antioxidant and inhibitor of melanogenesis.

## 1. Introduction

Melanin is important for skin color and plays a critical role in protecting the skin from ultraviolet (UV) light-induced damage. UV-induced skin hyperpigmentation results from abnormal melanin production [1]. In the melanin biosynthesis pathway, tyrosinase is the rate-limiting enzyme involved in the hydroxylation of l-tyrosine to l-3,4-dihydroxyphenylalanine (l-DOPA). l-DOPA is then further oxidized to the corresponding *o*-quinone. It has been found that several skin hyperpigmentation disorders, such as freckles, melasma, age spots, and other hyperpigmentation syndromes, are the result of the abnormal accumulation or overproduction of melanin. Thus, many skin depigmenting chemicals, such as arbutin and kojic acid, act as tyrosinase inhibitors that have been applied as skin whitening products for the treatment or prevention of abnormal skin pigmentation. However, side effects have been caused by the chemical inhibitors of tyrosinase, such as the possible genotoxic effect caused by arbutin [2] and the pigmented contact dermatitis due to kojic acid [3]. Therefore, the search for a safe and effective skin depigmenting agent is still a goal of many studies.

Reactive oxygen species (ROS) and free radicals are associated with several diseases, such as inflammation [4] and age-related diseases [5]. ROS-induced damage on the skin and UV stress plays an important role in photoaging [6]. It has been found that antioxidants can interfere with oxidation processes by acting as free radical scavengers or ROS scavengers or by chelating oxidation-catalytic metals. Therefore, numerous natural antioxidants or antioxidant supplements have been used to reduce oxidative damage in the human body. However, some synthetic chemical antioxidants, such as *tert*-butyl hydroxyanisole (BHA) and *tert*-butyl hydroxytoluene (BHT), have been reported to show carcinogenic effects in humans [7]. Thus, numerous studies on plant-derived antioxidants have been conducted during the past decade. Moreover, it was found that melanogenesis produces hydrogen peroxide (H_2_O_2_) and ROS, which place melanocytes under high-grade oxidative stress. It is well known that some ROS scavengers and inhibitors of ROS generation may downregulate UV-induced melanin production [8]. Therefore, inhibitors of melanogenesis, antioxidants, and ROS scavengers have been increasingly applied to skin care products for the prevention or treatment of undesirable skin hyperpigmentation.

Plants of the *Acorus* species have fragrant leaves and rhizomes; they belong to the *Acoraceae *family. Those plants are found throughout India, Europe, Japan, Central and Southeast Asia. They are also grown in the wet zones in Taiwan due to their medicinal properties. The biological activities of the essential oils extracted from several* Acorus* species have been extensively studied. The essential oil from the rhizomes of *A. calamus* has been reported to show anthelmentic and acetylcholinesterase inhibitory activities [9, 10]. The essential oil from the rhizomes of* A. gramineus* has also been found to exhibit neuroprotection functions [11]. However, there are no reports on the biological activity or chemical composition of essential oil extracted from *A. macrospadiceus*. Recently, our laboratory searched for valuable plant essential oils with dermatological usefulness [12]. In this study, we aimed to examine the inhibitory effects of the essential oil extracted from the leaves of *A. macrospadiceus* on melanogenesis, evaluate the antioxidant capacity of the oil, and analyze its chemical composition by GC/MS. 

## 2. Materials and Methods

### 2.1. Plant Material and Extraction of Essential Oil

The leaves of *A. macrospadiceus* were collected between May and July 2011 and identified at the Taichung District Agricultural Research and Extension Station in Taiwan. Essential oil was isolated by the hydrodistillation of the leaves (2 kg) in a Clevenger-type apparatus at 100°C for 2 h. The essential oil was then collected in a sealed glass bottle and stored at 4°C in a refrigerator until analysis. In this study, the essential oil was diluted with dimethyl sulfoxide (DMSO). DMSO was also used as a negative control in the experiments.

### 2.2. Mushroom Tyrosinase Activity Assessment

To measure the potential inhibitory effects of *A. macrospadiceus* essential oil on mushroom tyrosinase, enzyme inhibition experiments were performed in triplicate. In brief, 20 *μ*L of an aqueous solution of mushroom tyrosinase (200 units) was added to a 96-well microplate filled with 200 *μ*L of a mixture of 5 mM l-DOPA, 50 mM phosphate buffer (pH 6.8), and *A. macrospadiceus* essential oil (0.4, 0.8 and 2.0 mg/mL) or kojic acid (0.2 mM). The assay mixture was incubated at 37°C for 30 min. Following incubation, the amount of dopachrome produced in the reaction mixture was determined by the spectrophotometric analysis of absorbance at 490 nm.

### 2.3. Cell Culture and Cell Viability Assay

To evaluate the effect of* A. macrospadiceus* essential oil on B16F10 cell viability, the cell viability assay was performed in triplicate. B16F10 cells (ATCC CRL-6475; BCRC60031) were obtained from the Bioresource Collection and Research Center (BCRC) at the Food Industry Research and Development Institute in Hsinchu, Taiwan. The cells were cultured in Dulbecco's modification of Eagle's medium (DMEM) with 10% fetal bovine serum (FBS; Gibco, Langley, OK, USA) and 100 I.U/50 *μ*g/mL of penicillin/streptomycin (Sigma Chemical Co, Saint Louis, MO, USA) in a humidified atmosphere containing 5% CO_2_ in air at 37°C. The assay was performed using 3-(4,5-dimethylthiazol-2-yl)-2,5-diphenyltetrazolium bromide (MTT). The cells (1 × 10^4^ cells/well) were seeded into a 96-well plate and then exposed to the essential oil (0.4, 0.8, 1.6, and 3.2 mg/mL) for 24 h, followed by the addition of MTT solution to each well. The insoluble derivative of MTT produced by the intracellular dehydrogenase was solubilized with ethanol-DMSO (1 : 1 mixture solution). The absorbance of each well at 570 nm was measured using a microplate reader. The amount of MTT in the essential oil treated group was compared with the control group. Higher relative amounts of MTT indicate that the essential oil is not cytotoxic to the B16F10 cells.

### 2.4. Melanin Content Measurement

The B16F10 cells were first stimulated with alpha-melanocyte stimulating hormone (*α*-MSH) (100 nM) for 24 h and then further treated with either* A. macrospadiceus* essential oil (0.4, 0.8, and 2.0 mg/mL) or arbutin (2.0 mM) for an additional 24 h. After the treatments, the cells were detached by incubation in trypsin/EDTA and subsequently centrifuged at 5,000 g for 5 min. The cell pellets were then solubilized in 1 N NaOH at 60°C for 60 min. The melanin content was assayed by spectrophotometric analysis at 405 nm absorbance.

### 2.5. Intracellular Tyrosinase Activity Assay

The cells were first treated with *α*-MSH (100 nM) for 24 h and then further treated with various concentrations of* A. macrospadiceus* essential oil (0.4, 0.8, and 2.0 mg/mL) or arbutin (2.0 mM) for another 24 h. After the treatments, the cells were washed twice with phosphate-buffered saline (PBS) and homogenized with 50 mM PBS (pH 7.5) buffer containing 1.0% Triton X-100 and 0.1 mM phenylmethanesulfonyl fluoride (PMSF). Cellular extracts (100 *μ*L) were mixed with freshly prepared L-DOPA solution (0.1% in phosphate-buffered saline) and incubated at 37°C for 30 min. The absorbance at 490 nm was measured with a microplate reader Gen 5 (BIO-TEK Instrument, Winooski, VT, USA) to monitor the production of dopachrome.

### 2.6. DPPH Scavenging Activity Assay

The antioxidant activity of *A. macrospadiceus* essential oil was first determined by measuring the DPPH scavenging ability. The essential oil at various concentrations (0.045, 0.225, and 0.45 mg/mL) was added to 2.9 mL of the DPPH (60 *μ*M) solution. When DPPH reacts with an antioxidant that can donate hydrogen, DPPH is reduced, and the resulting decrease in absorbance at 517 nm is recorded using a UV-Vis spectrophotometer (Jasco, V-630, Tokyo, Japan). In this study, vitamin C (0.53 mg/mL) and BHA (0.1 mg/mL) were used as positive antioxidant standards.

### 2.7. ABTS^+^ Scavenging Capacity Assay

The ABTS decolorization assays were performed as previously described [12] and involves the generation of the ABTS^+^ chromophore through the oxidation of ABTS with potassium persulfate. The ABTS radical cation (ABTS^+^) was produced by reacting a 7 mM stock solution of ABTS with 2.45 mM potassium persulfate and allowing the mixture to stand in the dark for at least 6 h before use. Absorbance at 734 nm was measured 10 min after the mixing of various concentrations of* A. macrospadiceus* essential oil (0.045, 0.225, and 0.45 mg/mL) with 1 mL ABTS^+^ solution. The ABTS^+^scavenging capacity of* A. macrospadiceus* essential oil was compared with that of Trolox (0.0125 or 0.125 mg/mL).

### 2.8. Determination of Reducing Power

The reducing power of the *A. macrospadiceus* essential oil was determined according to a method described in previous work [13]. Different concentrations of* A. macrospadiceus* essential oil (0.012, 0.06, 0.12 mg/mL), vitamin C (0.105 mg/mL), or BHA (0.1 mg/mL) were mixed with phosphate buffer (2.5 mL, 0.2 M, pH 6.6) and potassium ferricyanide (K_3_Fe(CN)_6_) (2.5 mL, 1% w/v). The mixture was incubated at 50°C for 20 min. A portion (2.5 mL) of trichloroacetic acid (10% w/v) was added to the mixture and then centrifuged at 1,000 g for 10 min. The upper layer of the solution (2.5 mL) was mixed with distilled water (2.5 mL) and FeCl_3_ (0.5 mL, 0.1% w/v), and the absorbance was measured at 700 nm with a UV-Vis spectrophotometer. A high absorbance of the reaction mixture indicated a greater reducing power of the test sample.

### 2.9. Measurement of Metal-Ion Chelating Capacity

The chelation of ferrous ions by *A. macrospadiceus* essential oil was described in a previous method [12]. Different concentrations of essential oil (0.02, 0.1, and 0.2 mg/mL) or ethylenediaminetetraacetic acid (EDTA) (0.05, 0.06, and 0.07 mg/mL) were added to a solution of 1 mM FeCl_2_ (0.05 mL). Then, 0.1 mL of ferrozine (1 mM) was added to the reaction mixture, and the mixture was made up to 1 mL with methanol and left to stand for 10 min at 25°C. The absorbance of the reaction mixture was measured at 562 nm. The percentage of chelating capacity was calculated as follows:

(1) $$\begin{array}{c} \text{chelating}\;\text{capacity}\;\%\;=\left[\frac{\left(A_{1}-A_{2}\right)}{A_{1}}\times 100\right], \end{array}$$

where *A*_1_ is the absorbance of the control, and *A*_2_ is the absorbance in the presence of essential oil or EDTA.

### 2.10. Determination of Intracellular ROS Level

The B16F10 melanoma cells were cultured in 24-well plates (5 × 10^4^ cells in 1 mL of DMEM medium) and treated with various concentrations of *A. macrospadiceus* essential oil (0.4, 0.8, and 2.0 mg/mL) or Trolox (2.0 mM) for 24 h. The cells were then incubated with 24 mM H_2_O_2_ at 37°C for 30 min. After incubation, 2′,7′-dichlorofluorescein diacetate (DCFH-DA) was added to the wells, and the cells were cultured for 30 min. After treatment, the cells were washed with phosphate-buffered saline and trypsinized with trypsin/EDTA. The fluorescence intensities of DCF were measured at an excitation wavelength of 504 nm and emission wavelength of 524 nm using a fluorescent reader, Fluoroskan Ascent (Thermo Scientific, Vantaa, Finland). The data were analyzed with Ascent software (Thermo Scientific, Vantaa, Finland). Cells with increased level of ROS appeared as a population with high fluorescence intensity [14].

### 2.11. Gas Chromatography-Mass Spectrometry (GC-MS)

Analysis of the volatile chemical components in *A. macrospadiceus* essential oil was carried out using a Thermo GC-MS system (GC-MS Trace DSQ-Mass Spectrometer, MSD 201351, Thermo, Minneapolis, MN, USA). The Equity^TM-5^ capillary column (Supelco, St. Louis, MO, USA) with a 30-m length and 0.25-mm inner diameter was used with a 0.25-*μ*m-thick film. The oven temperature gradient was programmed as follows: isothermal at 40°C, followed by a 5°C temperature ramp every minute to 100°C, which was held for 5 minutes, followed by a temperature increase of 5°C every minute to 250°C, which was maintained for 20 minutes. The carrier gas was helium (1 mL/min). The injection port temperature was 250°C, and the detector temperature was also 250°C. Ionization of the essential oil (1 *μ*L) was performed in the EI mode (70 eV). The linear retention indices for all the compounds were determined by coinjection of the essential oil with a solution containing a homologous series of C8-C22 n-alkanes [15]. The individual components were identified by the retention indices and compared with compounds known from the literature [16]. Their mass spectra were also compared with known, previously obtained compounds or from the Trace DSQ-MASS spectral database (Thermo, USA).

### 2.12. Statistical Analysis

Statistical analysis of the experimental data points was performed using the one-way ANOVA test, which compared measured data using SPSS 12.0 statistical software (4th Edition, SPSS INC. Chicago, USA, 2007). The data are presented as the mean ± standard deviation of triplicate experiments. Differences were considered to be statistically significant when the *P* value was less than 0.05. 

## 3. Results

**Figure 1:**
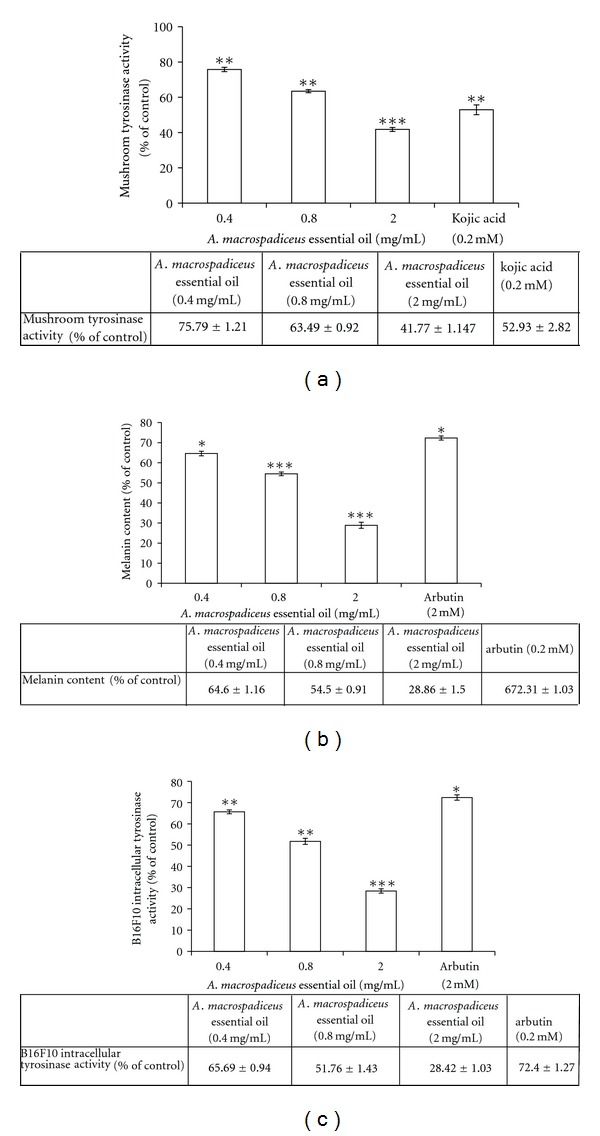
(a) Inhibitory effect of *A. macrospadiceus* essential oil on mushroom tyrosinase activity. (b) Effect of *A. macrospadiceus* essential oil on melanin synthesis in B16F10 cells. (c) Effect of *A. macrospadiceus* essential oil on tyrosinase activity in B16F10 cells. The results are expressed as percentages of the control. The data are presented as the mean ± SD for the three separate experiments. Values are significantly different compared with the control. *P < 0.05, **P < 0.01, ***P < 0.001.

**Figure 2:**
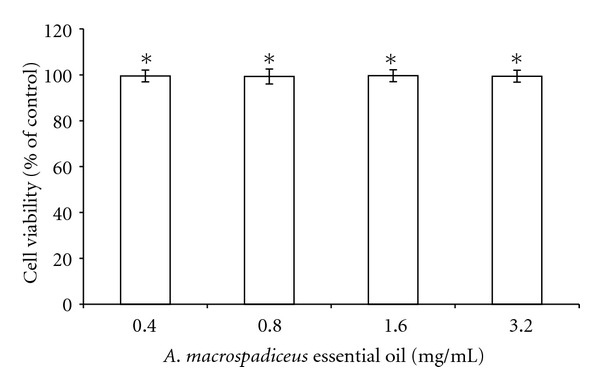
Cell viability assay. Results are expressed as the percentage of cell viability relative to the control. Data are presented as the mean ± S.D. Values are significantly different compared with the control. *P < 0.05.

*A. macrospadiceus* essential oil (0.4, 0.8, and 2.0 mg/mL) effectively inhibited mushroom tyrosinase activity. The residual tyrosinase activity was 75.79 ± 1.21%, 63.49 ± 0.92%, and 41.77 ± 1.147% of the control for 0.4, 0.8, and 2.0 (mg/mL) of essential oil, respectively. The IC_50_ of the essential oil was 1.57 mg/mL. Moreover, the mushroom tyrosinase activity was inhibited by the positive standard kojic acid (0.2 mM); the remaining enzyme activity was 52.93 ± 2.82% of that of the control. The highest concentration of the essential oil (2.0 mg/mL) exhibited a similar inhibitory effect on mushroom tyrosinase activity as kojic acid (Figure 1(a)). To further measure the possible inhibitory effect of *A. macrospadiceus* essential oil on melanin production, the melanin content in B16F10 melanoma cells was measured after treatment with different concentrations of essential oil or arbutin. The essential oil exhibited a significant inhibitory effect on melanin synthesis. After treatment, the melanin content was 64.6 ± 1.16%, 54.5 ± 0.91%, and 28.86 ± 1.56% for 0.4, 0.8, and 2.0 mg/mL essential oil, respectively. The IC_50_ of the essential oil was 1.04 mg/mL. Arbutin (2.0 mM) was used as a positive standard, and the residual intracellular melanin content for arbutin was 72.31 ± 1.03% of control (Figure 1(b)). The residual intracellular tyrosinase activity was 65.69 ± 0.94%, 51.76 ± 1.43%, and 28.42 ± 1.03% for 0.4, 0.8, and 2.0 mg/mL essential oil, respectively. The IC_50_ of the essential oil was 1.01 mg/mL. The intracellular tyrosinase activity was 72.4 ± 1.27% in the arbutin treated cells (Figure 1(c)). Furthermore, results of the MTT assay confirmed that the oil is not cytotoxic to the B16F10 cells at the dosages employed (0.4–3.2 mg/mL) (Figure 2). 

**Figure 3:**
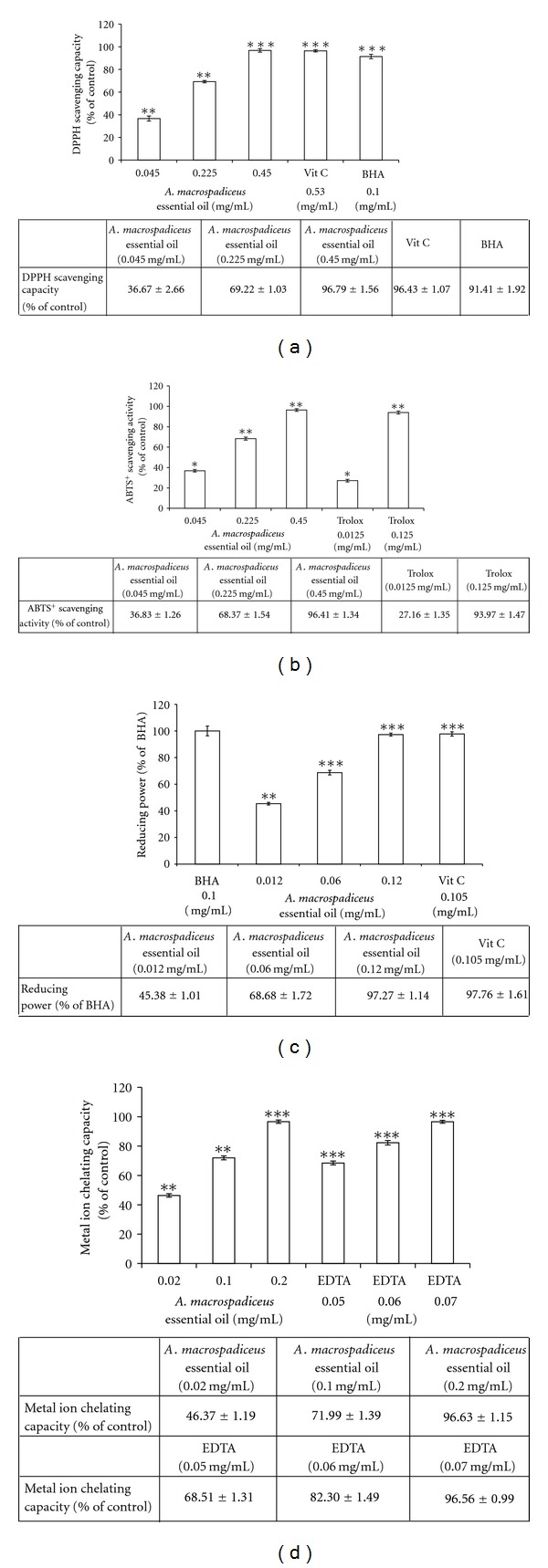
(a) DPPH radical scavenging capacity of *A. macrospadiceus* essential oil. (b) ABTS^+^ radical scavenging ability of *A. macrospadiceus* essential oil. (c) Reducing power of *A. macrospadiceus* essential oil. (d) Metal-ion chelating activity of *A. macrospadiceus* essential oil. Results are expressed as percentages of the control, and the data are presented as the mean ± SD for the three separate experiments. Values are significantly different compared with the control. *P < 0.05, **P < 0.01, ***P < 0.001.

**Figure 4:**
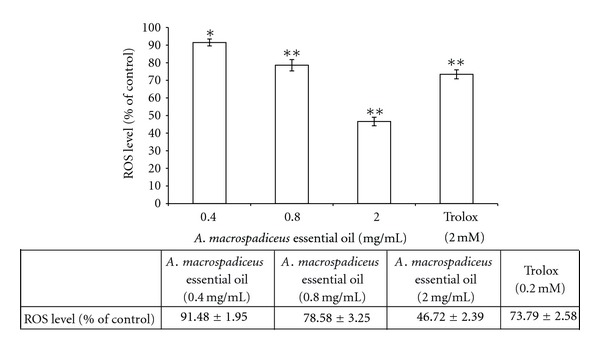
Effect of *A. macrospadiceus* essential oil on the B16F10 ROS level. Results are expressed as the percentage relative to the control. Data are presented as the mean ± S.D. Values are significantly different compared with the control. *P < 0.05, **P < 0.01.

The antioxidant capacity of *A. macrospadiceus* essential oil was first measured by measuring its DPPH scavenging ability. The DPPH scavenging activities of *A. macrospadiceus* essential oil, shown in Figure 3(a), are 36.67 ± 2.66%, 69.22 ± 1.03%, and 96.79 ± 1.56% of the control for 0.045, 0.225, and 0.45 mg/mL oil, respectively. The EC_50_ of the essential oil was 0.121 mg/mL. In addition, the radical scavenging activity was 96.43 ± 1.07% for vitamin C and 91.41 ± 1.92% for BHA. The ABTS^+^ assay was further employed to confirm the antioxidant activity of the essential oil. The ABTS^+^ scavenging capacity of the essential oil was 36.83 ± 1.26%, 68.37 ± 1.54%, and 96.41 ± 1.34% of the control for essential oil dosages of 0.045, 0.225, and 0.45 mg/mL, respectively. The EC_50_ of the essential oil was 0.122 mg/mL. The ABTS^+^ scavenging capacity of Trolox was 27.16 ± 1.35% and 93.97 ± 1.47% for 0.0125 mg/mL and 0.125 mg/mL Trolox, respectively (Figure 3(b)). The results, shown in Figure 3(c), reveal that higher concentrations of the essential oil have higher reducing powers; the reducing powers of 0.012, 0.06, and 0.12 mg/mL *A. macrospadiceus* essential oil were 45.38 ± 1.01%, 68.68 ± 1.72%, and 97.27 ± 1.14%, respectively. The reducing power of the essential oil with a dosage of 0.12 mg/mL was similar to that of vitamin C (0.105 mg/mL), which further confirms the antioxidant capacity of the oil. The metal-ion chelating abilities of 0.02, 0.1, and 0.2 mg/mL *A. macrospadiceus* essential oil were 46.37 ± 1.19%, 71.99 ± 1.39%, and 96.63 ± 1.15% of the control, respectively. In comparison, the metal-ion chelating capacities of 0.05, 0.06, and 0.07 mg/mL EDTA were 68.51 ± 1.31%, 82.30 ± 1.49%, and 96.56 ± 0.99%, respectively (Figure 3(d)). To further confirm the ability of *A. macrospadiceus* essential oil to scavenge free radicals in a cellular environment, the evaluation of intracellular ROS levels was conducted. In this assay, B16F10 cells were treated with 0.4, 0.8, and 2.0 mg/mL oil and Trolox (2.0 mM); the residual cellular ROS level were 91.48 ± 1.95%, 78.58 ± 3.25%, and 46.72 ± 2.39% for 0.4, 0.8, and 2.0 mg/mL oil, respectively. The residual cellular ROS level of the Trolox treated group was 73.79 ± 2.58% of the control (Figure 4). 

The chemical composition of* A. macrospadiceus* essential oil was analyzed by GC-MS, as shown in Table 1. The major component in the oil is methylchavicol (54.01%). Another kind of ether present in the oil is isosafrole (1.72%). Ketones comprise 19.57% of the oil, including nootkatone (15.92%) and isomenthone (3.64%). The only monoterpene in the essential oil is limonene (7.82%). The oil contains two types of alcohols, namely, linalool (3%) and (−)-menthol (0.85%). There are three types of esters in the essential oil, namely, linalyl formate (2.57%), nerol acetate (0.68%) and geranyl acetate (0.52%). Eremophilene (3.39%) and **τ**-selinene (0.33) are the two sesquiterpenes present in the oil. The only aromatic compound in the oil is *o*-cymene (2.85%). However, 2.69% of the compounds are unknown (Table 1). 

## 4. Discussion

Mushroom tyrosinase is commonly used as the target enzyme in screening possible inhibitors of melanin production. It has been reported that the methanol extract of *A. gramineus *inhibited mushroom tyrosinase activity and decreased the melanin content in B16F10 cells [17]. However, there is no report on the dermatological application of essential oils extracted from plants belonging to the *Acorus *family. The results of this study show that essential oil extracted from the leaves of *A. macrospadiceus* significantly inhibits mushroom tyrosinase; thus, the essential oil may act as a possible tyrosinase inhibitor. It has been found that kojic acid is not effective in inhibiting melanin production or cellular tyrosinase in B16F10 cells [12, 13]. Thus, arbutin was selected as a positive standard in the following evaluation of intracellular melanin content and tyrosinase activity in B16F10 cells. The results show in Figure 1(b), that essential oil extracted from leaves of* A. macrospadiceus* has a stronger inhibitory effect on melanin formation in B16F10 cells than arbutin. We further assessed the intracellular tyrosinase activity in B16F10 cells after the same treatment with essential oil or arbutin. It was found that the essential oil significantly inhibited *α*-MSH-induced tyrosinase activity. The results shown in Figure 1(b) indicate that the essential oil extracted from leaves of* A. macrospadiceus* show a stronger inhibitory effect on melanin formation in B16F10 cells than arbutin. Furthermore, we found that the essential oil effectively inhibits B16F10 intracellular tyrosinase activity. The results shown in Figure 1(c) agree with the results in Figure 1(b), which means that the essential oil inhibited B16F10 intracellular tyrosinase activity and decreased melanin content. This shows the considerable depigmentation potential of *A. macrospadiceus* essential oil in the B16F10 cell model. In these experiments, *α*-MSH acted as a cyclic-3′, 5′-adenosine monophosphate (cAMP) inducer to stimulate melanin synthesis. It has been reported that *α*-MSH can bind the melanocortin 1 receptor (MC1R) and activate adenylate cyclase, which in turn catalyzes ATP to cAMP and increases the intracellular cAMP level [18]. In the present study, the results show that* A. macrospadiceus* essential oil inhibited B16F10 melanogenesis induced by *α*-MSH mediated intracellular cAMP upregulation. The MTT assay is a simple and rapid method of screening for the drug responsiveness of cell lines; results revealed that the oil has no inhibitory effect on B16F10 cell growth. All these results indicate that the inhibitory effects of *A. macrospadiceus* essential oil on melanogenesis in B16F10 cells were not the results from cell death (Figure 2).

The different free radical scavenging activities of the essential oil against DPPH and ABTS^+^ radicals may be the result of different mechanisms of the antioxidant-radical interactions in the two assays. Additionally, the stoichiometry of the reactions between the potential antioxidant chemicals in the essential oil may be different, which resulted in the difference in radical scavenging capacity [19]. To measure the reducing power of *A. macrospadiceus* essential oil, various concentrations of the essential oil (0.012, 0.06, 0.12 mg/mL) and vitamin C (0.105 mg/mL) were tested. BHA (0.1 mg/mL) was used as a positive standard. The conversion of a Fe^3+^/ferricyanide complex to the ferrous form serves as an indicator of antioxidant capacity. Antioxidants may interact with ferrous ions to form insoluble metal complexes and then inhibit the interaction between the metal and lipid. The metal-ion chelating capacity of *A. macrospadiceus* essential oil increased with increasing concentrations of the oil. It was further confirmed that *A. macrospadiceus* essential oil exactly has a potent antioxidant capacity. Although chemical tests to assess antioxidant activity are often used, the best approach to study the antioxidant activity of a given compound would be directly *in vivo* or in a cellular model. Many compounds that provide an antioxidant effect in vitro are pro-oxidant when tested on cells [20]. In fact, antioxidant compounds have different means for exerting their action *in vivo*, including interactions with intracellular signal transduction pathways or inducing the expression of antioxidant and detoxification enzymes. The principle of the DCFH-DA assay is that DCFH-DA could diffuse through the cell membrane and become enzymatically hydrolyzed by esterase to DCFH, which in turn reacts with ROS (such as H_2_O_2_) to yield DCF. Rapid increases in DCF indicate the oxidation of DCFH by intracellular radicals. Our results show that *A. macrospadiceus* essential oil effectively depleted the intracellular ROS level, which places the cells under a lower grade of oxidative stress (Figure 4). It is very important to make sure that products that are applied topically on the skin should not increase the oxidative stress of skin fibroblasts or melanocytes. 

Human skin exposed to UV light or environmental oxidizing pollutants become a preferred target of oxidative stress. It has been proved that UV irradiation induces ROS generation in cutaneous tissue, provoking damages, such as enzyme inactivation and lipid peroxidation. To counteract oxidative damage, the skin is equipped with a network of enzymatic and nonenzymatic antioxidant systems. *A. macrospadiceus* essential oil has shown considerable antioxidant potential based on the DPPH, ABTS^+^ radical scavenging, reducing power, and metal-chelating analytical studies. The results exhibited a dose-dependent increase in antioxidant potential over different ranges with distinct efficiencies. Natural antioxidants have gained increasing interest among consumers and the research community because epidemiological studies have indicated that frequent consumption of natural antioxidants is associated with a lower risk of cancer, cardiovascular, and neurodegenerative diseases [21]. Essential oils are a source of natural antioxidants and have been reported to show beneficial effects on human health [22]. To date, there are no reports on the benefits of *A. macrospadiceus* essential oil on skin health. 

The search for antioxidants with skin-depigmenting capabilities is driven by the hypothesis that oxidative stress resulting from UV-irradiation may contribute to the stimulation of melanin production. It has been reported that UV irradiation can produce ROS in the cutaneous tissues that may induce melanin synthesis by activating tyrosinase, as the enzyme prefers the superoxide anion radical (O_2_^−^) over the oxygen molecule (O_2_) [23]. In addition, it was found that redox agents can also influence melanin production by interacting with the copper ion at the active site of tyrosinase or with *o*-quinones to block the oxidative polymerization of melanin intermediates [24]. Moreover, some antioxidants, such as vitamin B, vitamin C, or vitamin E, can also reduce the photooxidation of preexisting melanin particles. Thus, these vitamins with antioxidant activities are commonly applied in skin-whitening cosmetic formulations [25]. Our research indicates that the apparent antioxidant capacity of *A. macrospadiceus* essential oil could be applied in cosmetic formulations of skin care products. 

The GC-MS data revealed the presence of seven types of chemical components in *A. macrospadiceus* essential oil, summarized in Table 1, namely, ethers (55.73%), ketones (19.57%), monoterpene (7.82%), alcohols (3.85%), esters (3.77%), sesquiterpenes (3.72%), and aromatic compound (2.85%). These compounds account for 97.31% of the essential oil. An earlier study on the volatiles from the leaves and rhizomes of *Acorus* spp. found that methylchavicol (49%) is the major constituent of volatiles from the leaves (48.8%) and rhizomes (41.4%) of *Acorus gramineus* var. “liquorice” [26]. In the present study, methylchavicol (54.01%) is the major constituent of *A. macrospadiceus* essential oil and imparts the characteristic anisic odor of this plant. However, the other chemical components in this essential oil were different from those of the other *Acorus* species [26]. Nootkatone (15.92%) is the major ketone in this essential oil. Nootkatone is reported to show an inhibitory effect on rat platelet aggregation [27]. Future research should include the effect of nootkatone on tyrosinase or melanin production. The only aromatic compound, *o*-cymene (2.85%), may have accounted for the antioxidant properties of the essential oil [28]. Some synthetic ethers have been reported to show antioxidant activities [29]; thus, we assume that methylchavicol and isosafrole may have contributed to the antioxidant activity of the essential oil extracted from the leaves of* A. macrospadiceus*.

## 5. Conclusion

This is the first report on the inhibitory effect of essential oil extracted from the leaves of *A. macrospadiceus* on melanin synthesis. We also analyzed the antioxidant capacities and chemical composition of the essential oil. The present study concluded that *A. macrospadiceus* essential oil inhibits melanin synthesis in B16F10 melanoma cells and showed antioxidant potential. The results indicate that the decrease in melanin production may be attributed to the oil's inhibitory action on the signaling pathway regulating tyrosinase activity or the depletion of cellular oxidative stress. The essential oil can thereby serve as an inhibitor of melanin production, which could also act as a natural antioxidant. Our research showed that essential oils extracted from leaves of *A. macrospadiceus* could be applied in the cosmetic formulation of skin care products. 

## Figures and Tables

**Table 1:** Chemical composition of the essential oil from the leaves of *A*. *macrospadiceus*.

*R*_*t*_^a^	Compound^b^	M. f.^c^	Peak area (%)	Classification
27.25	*o*-Cymene	C_10_H_14_	2.85	Aromatic compound
27.47	Limonene	C_10_H_16_	7.82	Monoterpene
30.94	Linalool	C_10_H_18_O	3.00	Alcohol
33.03	Isomenthone	C_10_H_18_O	3.64	Ketone
33.97	(−)-Menthol	C_10_H_20_O	0.85	Alcohol
35.28	Chavicol methyl ether	C_10_H_12_O	54.01	Ether
39.04	Linalyl formate	C_11_H_18_O_2_	2.57	Ester
46.54	Nerol acetate	C_12_H_20_O_2_	0.68	Ester
47.48	Geranyl acetate	C_12_H_20_O_2_	0.52	Ester
51.39	**τ**-Selinene	C_15_H_24_	0.33	Sesquiterpene
51.68	Eremophilene	C_15_H_24_	3.39	Sesquiterpene
53.80	Isosafrole	C_10_H_10_O_2_	1.72	Ether
58.28	Nootkatone	C_15_H_22_O	15.92	Ketone
	Unknown		2.69	

^a^ *R*_*t*_: Retention time (min). ^b^The components were identified by their mass spectra and retention indicates (RIs) of the Wiley and NIST mass spectral databases and previously published RIs. ^c^M. f.: Molecular formula.

## Abbreviations

ABTS: 2, 2′-azino-bis (3-ethylbenzthiazoline-6-sulphonic acid)
*α*-MSH: Alpha-melanocyte stimulating hormone
BHA: Tert-butyl hydroxyanisole
BHT: Tert-butyl hydroxytoluene
cAMP: Cyclic-3′, 5′-adenosine monophosphate
DCFH-DA: 2′, 7′-dichlorofluorescein diacetate
DMEM: Dulbecco's modification of Eagle's medium
DMSO: Dimethyl sulfoxide
DPPH: 2, 2-diphenyl-1-picryl-hydrazyl
DSQ: Detection system quadrupole
EDTA: Ethylenediaminetetraacetic acid
EI: Electron impact
FBS: Fetal bovine serum
GC/MS: Gas chromatography/mass spectrometry
l-DOPA: l-3, 4-dihydroxyphenylalanine
MC1R: Melanocortin 1 receptor
MTT: 3-(4, 5-dimethylthiazol-2-yl)-2, 5-diphenyltetrazolium bromide
PBS: Phosphate-buffered saline
PMSF: Phenylmethanesulfonyl fluoride
ROS: Reactive oxygen species
UV: Ultraviolet.
